# Tunneling times of acoustic phonon packets through a distributed Bragg reflector

**DOI:** 10.1186/1556-276X-9-449

**Published:** 2014-08-29

**Authors:** Zorayda Lazcano, Pedro Luis Valdés Negrín, Diosdado Villegas, Jesus Arriaga, Rolando Pérez-Álvarez

**Affiliations:** 1 Instituto de Física, Benemérita Universidad Autónoma de Puebla, Apartado Postal J-48, CP 7250 Puebla, México; 2 Departamento de Física, Universidad Central ‘Marta Abreu’ de Las Villas, CP 54830 Santa Clara, Cuba; 3 Universidad Autónoma del Estado de Morelos, Ave. Universidad 1001, CP 62209 Cuernavaca, México

**Keywords:** Distributed Bragg reflector, Tunneling time, Hartman effect

## Abstract

The longwave phenomenological model is used to make simple and precise calculations of various physical quantities such as the vibrational energy density, the vibrational energy, the relative mechanical displacement, and the one-dimensional stress tensor of a porous silicon distributed Bragg reflector. From general principles such as invariance under time reversal, invariance under space reflection, and conservation of energy density flux, the equivalence of the tunneling times for both transmission and reflection is demonstrated. Here, we study the tunneling times of acoustic phonon packets through a distributed Bragg reflector in porous silicon multilayer structures, and we report the possibility that a phenomenon called Hartman effect appears in these structures.

## Background

Phonons, the quanta of lattice vibrations, manifest themselves practically in all electrical, thermal, and optical phenomena in semiconductors and other material systems. The reduction of the size of electronic devices below the acoustic phonon mean free path creates a new situation for phonon propagation and interaction, opening up an exciting opportunity for engineering phonon spectrum in nanostructured materials [1]. Since the early work of Narayanamurti et al. [2], important progress has lately emerged in the development of nanowave phononic devices including, e.g., mirrors, cavities, and monochromatic sources.

How long does it take for a particle to tunnel through a potential barrier? This is a question that has occupied physicists for decades and one for which there is still no definitive answer [3]. The Hartman effect (HE) states that the tunneling time becomes independent of the barrier length [4]. The independence of tunneling time on barrier length would imply arbitrarily large and indeed superluminal velocities for tunneling wave packets, if tunneling was in fact a propagation phenomenon.

Phonon tunneling studies have also revealed phenomena related to the HE. Recent experiments on tunneling acoustic waves have reported the breaking sound barrier [5,6]. Yang et al. found that inside a phononic band, the group velocity increases linearly with the sample thickness, a rather remarkable effect that is a signature of tunneling in quantum mechanics [6,7]. Villegas et al. have discussed the physical conditions under which the tunneling time for long-wavelength phonons through semiconductor heterostructures is independent on the system’s size, i.e., the effect equivalent to the HE for electrons [8]. Experimental studies of HE in the context of the nanophononics have been carried out [9,10]. In these works, very short transit times in the stop bands have been measured, one acoustic equivalent of HE of electron tunneling through potential barriers.

During the last decade, interest in achieving all-silicon-based opto- and microelectronics was highly stimulated by the discovery of the unique optical properties of porous silicon [11]. Porous silicon is known as a versatile material with applications in light emission, sensing, and photonic crystal devices. It is well known that the introduction of artificial spatial periodicity in the elastic properties of a system results in Brillouin zone folding. Such folding is often accompanied by the appearance of bandgaps in the phonon frequency spectrum [12,13]. In the last few years, this interest has been translated to porous silicon-based phononic systems [14-17]. Here, we study the tunneling times of acoustic phonon packets through a distributed Bragg reflector in porous silicon multilayer structures.

The paper is organized as follows: The ‘Methods’ section provides some fundamentals in both the long-wavelength model and the transfer matrix method. The main theoretical findings are presented in the ‘Results and discussion’ section. In particular in this section, from general principles such as invariance under time reversal, invariance under space reflection, and conservation of energy density flux, the equivalence of the tunneling times for both transmission and reflection is demonstrated. At the end of the paper, the main conclusions are given.

## Methods

### Long-wavelength model

The one-dimensional energy density [18] is defined as

(1) $$ℋ=\frac{1}{2}\rho {\left|\frac{\mathrm{\partial u}}{\mathrm{\partial t}}\right|}^{2}+\frac{1}{2}\rho \omega_{\Gamma }^{2}|u{|}^{2}+\frac{1}{4}\left[\sigma {\frac{\mathrm{\partial u}}{\mathrm{\partial z}}}^{*}+\sigma^{*}\frac{\mathrm{\partial u}}{\mathrm{\partial z}}\right],$$

where the first term in (1) represents the kinetic energy density, the second one represents the interaction energy density of the phonon field with itself, and the third one represents the strain energy density that accounts for the dispersive character of the oscillations. These terms depend on the atomic relative displacements *u*, the linear mass density *ρ*, the phonon frequency at the center of the Brillouin zone *ω*_*Γ*_, the one-dimensional strain tensor *∂u*/*∂z*, and the stress tensor *σ*, which is equal to

(2) $$\sigma =-\rho \beta^{2}\mathrm{\partial u}/\mathrm{\partial z},$$

being *β* a parameter that accounts on the behavior of the bulk phonon dispersion relation. From Equations 1 and 2, we can obtain the one-dimensional equations of motion,

(3) $$\frac{\partial^{2}u}{\partial t^{2}}=-\omega_{\Gamma }^{2}u-\beta^{2}\frac{\partial^{2}u}{\partial z^{2}},$$

and

(4) $$\frac{\partial^{2}\sigma }{\partial t^{2}}=-\omega_{\Gamma }^{2}\sigma -\beta^{2}\frac{\partial^{2}\sigma }{\partial z^{2}}.$$

The continuity equation for the energy density is defined as

(5) ∂ℋ/∂t+∂j/∂z=0,

where the energy density flux, *j*, is given by

(6) $$j=-1/2\left(\sigma \partial u^{*}/\mathrm{\partial t}+\sigma^{*}\mathrm{\partial u}/\mathrm{\partial t}\right).$$

By substituting Equation 2 into Equation 6, we obtain the following expression for the energy density flux:

(7) $$j=\frac{\rho \beta^{2}}{2}\left(\frac{\partial u^{*}}{\mathrm{\partial t}}\frac{\mathrm{\partial u}}{\mathrm{\partial z}}+\frac{\mathrm{\partial u}}{\mathrm{\partial t}}\frac{\partial u^{*}}{\mathrm{\partial z}}\right).$$

A very convenient method for calculating the propagation of harmonic waves through a system consisting of a finite number of uniform layers is the transfer matrix method [19,20]. The transfer matrix, which propagates the wave amplitudes across a *j*-th homogeneous layer is given by

(8) $$M_{j}=\left(\begin{array}{cc} \cos\left(k_{j}d_{j}\right) & -\frac{1}{\omega Z_{j}}\sin\left(k_{j}d_{j}\right)\\ \omega Z_{j}\sin\left(k_{j}d_{j}\right) & \cos\left(k_{j}d_{j}\right) \end{array}\right),$$

where *Z*_*j*_=*ρ*_*j*_*v*_*j*_ is the acoustic impedance, *ρ*_*j*_ the mass density, *v*_*j*_ the velocity of sound, and *d*_*j*_ the layer width.

## Results and discussion

### Equivalence of the transmission and reflection times in the tunneling of long-wavelength phonons

**Figure 1:**
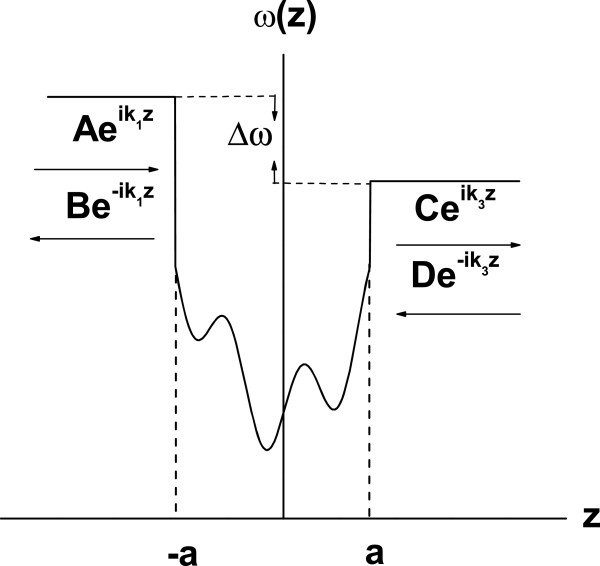
**General stationary scattering configuration.** The general stationary scattering configuration in one dimension. An arbitrary barrier is confined to the interval (−*a*,*a*).

In this paper, for the reason of complement, we give a demonstration of the equivalence of the transmission and reflection times (*τ*_t_=*τ*_r_) in the tunneling of long-wavelength phonons [21]. Consider the general phonon scattering process as shown schematically in Figure 1. Let us consider that the phonon propagation is normal to the layer interfaces and adopt the continuum model valid for long-wavelength oscillations. In this section, we analyze the properties of the transfer matrix in the tunneling process of long-wavelength phonons.

#### Invariance under time reversal

Let us observe that if we take the complex conjugate of (3) and if *t* is replaced by −*t*, we get

(9) $$\frac{\partial^{2}}{\partial t^{2}}u^{*}(z,-t)=-\omega_{\Gamma }^{2}(z)u^{*}(z,-t)-\beta {\left(z\right)}^{2}\frac{\partial^{2}}{\partial z^{2}}u^{*}(z,-t),$$

provided only that *β*(*z*) and *ω*_*Γ*_(*z*) are real functions. Observe that this equation has the same form as (3). Therefore, if *u*(*z*,*t*) is a solution of (3), then *u*^∗^(*z*,−*t*) is also a solution. *u*^∗^(*z*,−*t*) is often referred as the time-reversed solution. The behavior of the wave equation exhibited by (9) is called invariance under time reversal. For the stationary state, invariance under time reversal implies that if *u*(*z*) is a stationary-state wave function, then *u*^∗^(*z*) is also one.

The general solution of the wave equation for the system depicted in Figure 1 is given by

(10) $$u_{1}(z)=\left\{\begin{array}{ll} Ae^{ik_{1}z}+Be^{-ik_{1}z} & \;\;z<-a\\ \Psi (z,k_{2}) & \;\;-a<z<a\\ Ce^{ik_{3}z}+De^{-ik_{3}z} & \;\;z>a \end{array}\right),$$

where $k_{1}=\sqrt{\left(\omega_{\Gamma_{1}}^{2}-\omega^{2}\right)/\beta_{1}^{2}}\;\left(k_{3}=\sqrt{\left(\omega_{\Gamma_{3}}^{2}-\omega^{2}\right)/\beta_{3}^{2}}\right)$ is the wave number in the left (right) part of the system. Using the transfer matrix, we can relate the wave amplitudes in the left and right parts according to

(11) $$\left(\begin{array}{c} A\\ B \end{array}\right)=M\left(\begin{array}{c} C\\ D \end{array}\right),$$

where

(12) $$M=\left(\begin{array}{ll} M_{11} & M_{12}\\ M_{21} & M_{22} \end{array}\right).$$

On the other hand, the time-reversed solution of the wave equation is

(13) $$u_{2}(z)=\left\{\begin{array}{ll} A^{*}e^{-ik_{1}z}+B^{*}e^{ik_{1}z} & \;\;z<-a\\ \Psi^{*}(z,k_{2}) & \;\;-a<z<a\\ C^{*}e^{-ik_{3}z}+D^{*}e^{ik_{3}}z & \;\;z>a \end{array}\right).$$

Hence, comparing Equations 10, 11, and 13, we get

(14) $$\left(\begin{array}{c} A\\ B \end{array}\right)={\tilde{M}}^{*}\left(\begin{array}{c} C\\ D \end{array}\right),$$

where

(15) $${\tilde{M}}^{*}=\left(\begin{array}{cc} M_{22}^{*} & M_{21}^{*}\\ M_{12}^{*} & M_{11}^{*} \end{array}\right),$$

The properties of the transfer matrix in the tunneling of long-wavelength phonons can be obtained from Equations 11 and 14

(16) $$M_{11}^{*}=M_{22}\;\text{and}\;M_{12}^{*}=M_{21}.$$

#### Conservation of energy density flux

To demonstrate the conservation of the energy density flux, we need to calculate this quantity in the left and right parts of the system. Using Equations 7 and 10, we obtain

(17) $$j^{\text{L}}=\rho_{1}\omega \beta_{1}^{2}\text{Im}\left[u_{1}^{*}(z)\frac{du_{1}(z)}{\text{dz}}\right]=-\omega {\tilde{Z}}_{1}\left[|A{|}^{2}-|B{|}^{2}\right],$$

(18) $$j^{\text{R}}=\rho_{3}\omega \beta_{3}^{2}\text{Im}\left[u_{1}^{*}(z)\frac{du_{1}(z)}{\text{dz}}\right]=-\omega {\tilde{Z}}_{3}\left[|C{|}^{2}-|D{|}^{2}\right],$$

where the superscript L (R) refers to the left (right) of the region (−*a*,*a*), Im denote imaginary part and ${\tilde{Z}}_{1}=\rho_{1}\beta_{1}^{2}k_{1}$ and ${\tilde{Z}}_{3}=\rho_{3}\beta_{3}^{2}k_{3}$. The conservation of the energy density flux is expressed as *j*^L^=*j*^R^, or equivalently

(19) $$M^{t}\;\tilde{I}M^{*}=\frac{{\tilde{Z}}_{3}}{{\tilde{Z}}_{1}}\tilde{I},$$

where *M*^*t*^ and *M*^∗^ denote the transpose and conjugate matrix, respectively, and

(20) $$\tilde{I}=\left(\begin{array}{ll} 1 & 0\\ 0 & -1 \end{array}\right).$$

Using Equation 19 and the condition (16), one further condition on matrix *M* can be added

(21) $$\left|M\right|=M_{11}M_{22}-M_{12}M_{21}=\frac{{\tilde{Z}}_{3}}{{\tilde{Z}}_{1}}.$$

#### Invariance under space reflection

Now, let us consider the invariance under space reflection of Equation 3. Because *β*^2^(*z*), *ω*^2^(*z*), and $\omega_{\Gamma }^{2}(z)$ are even functions of *z*, another solution of the wave equation is obtained by replacing *z* by −*z*. We can immediately write the general solution of the wave equation as

(22) $$u_{3}(z)=\left\{\begin{array}{ll} Ae^{-ik_{1}z}+Be^{ik_{1}z} & \;\;z>a\\ \Psi (-z,k_{2}) & \;\;a>z>-a\\ Ce^{-ik_{3}z}+De^{ik_{3}z} & \;\;z<-a \end{array}\right),$$

and obtain the following relation

(23) $$\left(\begin{array}{c} C\\ D \end{array}\right)=\tilde{M}\left(\begin{array}{c} A\\ B \end{array}\right).$$

Substituting (23) into (14) gives us the condition

(24) $$M\tilde{M}=I,$$

where *I* is the identity matrix. From this last equation, we obtain

(25) $${\left|M_{11}\right|}^{2}+{\left|M_{12}\right|}^{2}=1\;\text{and}\;M_{11}(M_{12}+M_{12}^{*})=0.$$

The transmission (reflection) amplitude $A_{\text{t}}^{\text{R}}\left(A_{\text{r}}^{\text{R}}\right)$ for a phonon incident perpendicular to the barrier from right to left (the inverse process is described by *D*=1,*C*=*A*rR,*A*=0,*B*=*A*tR) and the transmission (reflection) amplitude $A_{\text{t}}^{\text{L}}\left(A_{\text{r}}^{\text{L}}\right)$ for a phonon incident from left to right (the direct process is described by $A=1,B=A_{\text{r}}^{\text{L}}$, $C=A_{\text{t}}^{\text{L}},D=0)$ are given by the following equations:

(26) $$\begin{array}{lcrr} A_{\text{t}}^{\text{R}}\left(k_{3},k_{1}\right)=\frac{\left|M\right|}{M_{11}}=\left|A_{\text{t}}^{\text{R}}\left(k_{3},k_{1}\right)\right|e^{i\alpha_{\text{t}}^{\text{R}}\left(k_{3},k_{1}\right)}, & & & \;\\ A_{\text{t}}^{\text{L}}\left(k_{1},k_{3}\right)=\frac{1}{M_{11}}=\left|A_{\text{t}}^{\text{L}}\left(k_{1},k_{3}\right)\right|e^{i\alpha_{\text{t}}^{\text{L}}\left(k_{1},k_{3}\right)}, \end{array}$$

(27) $$\begin{array}{lcrr} A_{\text{r}}^{\text{R}}\left(k_{3},k_{1}\right)=-\frac{M_{12}}{M_{11}}=\left|A_{\text{r}}^{\text{R}}\left(k_{3},k_{1}\right)\right|e^{i\beta_{\text{r}}^{\text{R}}\left(k_{3},k_{1}\right)}, & & & \;\\ A_{\text{r}}^{\text{L}}\left(k_{1},k_{3}\right)=\frac{M_{21}}{M_{11}}=\left|A_{\text{r}}^{\text{L}}\left(k_{1},k_{3}\right)\right|e^{i\beta_{\text{r}}^{\text{L}}\left(k_{1},k_{3}\right)}, \end{array}$$

in terms of the modulus and phase. By applying conditions (16) and (21) to the previous relations, we obtain

(28) $$A_{\text{t}}^{\text{R}}\left(k_{3},k_{1}\right)=\frac{\left|M\right|}{M_{11}}=\frac{{\tilde{Z}}_{3}}{{\tilde{Z}}_{1}}A_{\text{t}}^{\text{L}}\left(k_{1},k_{3}\right),$$

(29) $$A_{\text{r}}^{\text{R}}\left(k_{3},k_{1}\right)=-\frac{M_{12}}{M_{21}}A_{\text{r}}^{\text{L}}(k_{1},k_{3}).$$

Finally, the transmission and reflection coefficients are given by

(30) $$\begin{array}{lcll} T^{\text{R}}(k_{3})=\frac{j_{\text{t}}^{\text{R}}}{j_{\text{i}}^{\text{R}}} & = & \frac{{\tilde{Z}}_{1}}{{\tilde{Z}}_{3}}{\left|A_{\text{t}}^{\text{R}}(k_{3},k_{1})\right|}^{2}, & \;\\ T^{\text{L}}(k_{1})=\frac{j_{\text{t}}^{\text{L}}}{j_{\text{i}}^{\text{L}}} & = & \frac{{\tilde{Z}}_{3}}{{\tilde{Z}}_{1}}{\left|A_{\text{t}}^{\text{L}}(k_{1},k_{3})\right|}^{2}, & \;\\ R^{\text{R}}(k_{3})=\frac{j_{\text{r}}^{\text{R}}}{j_{\text{i}}^{\text{R}}} & = & {\left|A_{\text{r}}^{\text{R}}(k_{3},k_{1})\right|}^{2}, & \;\\ R^{\text{L}}(k_{1})=\frac{j_{\text{r}}^{\text{L}}}{j_{\text{i}}^{\text{L}}} & = & {\left|A_{\text{r}}^{\text{L}}(k_{1},k_{3})\right|}^{2}, & \; \end{array}$$

where t, r, and i denote transmitted, reflected, and incident, respectively. From Equations 16, 27, 28, and 30, it is very easy to prove that $T^{\text{R}}(k_{3})=T^{\text{L}}(k_{1})$ and $R^{\text{R}}(k_{3})=R^{\text{L}}(k_{1})$. From Equation 28, we obtain *α*tR(*k*_3_,*k*_1_)=*α*tL(*k*_1_,*k*_3_), and from Equations 16, 26, 27, 28, and 29, we can deduce the relation

(31) $$\beta_{\text{r}}^{\text{R}}(k_{3},k_{1})=\pm \pi -\beta_{\text{r}}^{\text{L}}(k_{1},k_{3})+2\alpha_{\text{t}}^{\text{L}}(k_{1},k_{3}).$$

If we consider that both materials in the left and right parts of Figure 1 are identical, i.e., (*k*_1_=*k*_3_≡*k*), then *α*tR(*k*_3_,*k*_1_)=*α*tL(*k*_1_,*k*_3_)≡*α*_t_(*k*) and *β*rR(*k*_3_,*k*_1_)=*β*rL(*k*_1_,*k*_3_)≡*β*_r_(*k*). By using this relations, the phase is

(32) $$\alpha_{\text{t}}(k)=\pm \pi /2+\beta_{\text{r}}(k).$$

Finally, using Equation 32 is straightforward to prove that the transmission and reflection phase times given by

(33) $$\tau_{\text{t}}=\frac{d\alpha_{\text{t}}}{\mathrm{d\omega}},$$

and

(34) $$\tau_{\text{r}}=\frac{d\beta_{\text{r}}}{\mathrm{d\omega}},$$

are equal,

(35) $$\tau_{\text{t}}=\tau_{\text{r}}.$$

Falck et al. have obtained a similar result for symmetric scattering potentials [22].

### Tunneling of acoustic phonons through a distributed Bragg reflector

**Figure 2:**
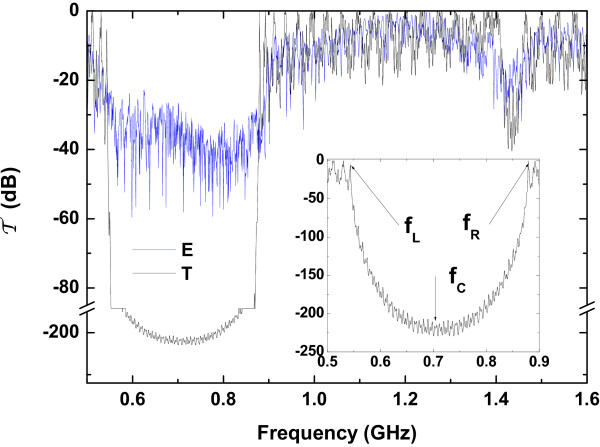
**Transmission coefficients.** Theoretical and experimental transmission coefficients (in dB) plotted as a function of the acoustic phonon frequencie (in GHz) for the DBR, with *N*=18. The inset shows some details of the theoretical transmission coefficient around the first acoustic stop band.

In this section, we apply the concepts discussed previously to study a distributed Bragg reflector (DBR) formed by a finite number of periods, *N*, based on porous silicon (PSi). The DBR is obtained by stacking periodically *N* times two constituent layers *A* and *B* with different porosity. The thicknesses of *A* and *B* layers are denoted by *d*_*A*_ and *d*_*B*_, respectively. In PSi, the mass density is related with the porosity *P* via *ρ*=*ρ*_0_(1−*P*), being *ρ*_0_ the mass density of bulk silicon. The propagating velocity of the longitudinal waves through PSi is related with the porosity as *v*=*v*_0_(1−*P*)^*k*^, being *v*_0_ the longitudinal velocity of the wave in bulk silicon, and *k* is a parameter. The acoustic impedance of layer *A*(*B*) is given by *Z*_*A*_=*ρ*_*A*_*v*_*A*_(*Z*_*B*_=*ρ*_*B*_*v*_*B*_). The dependence on porosity of these two parameters, velocity and mass density, requires a very accurate control of the etching process. We grow the layers forming the DBR according to the methodology reported in [23]. The thicknesses of the PSi layers are *d*_*A*_=2 *μ*m and *d*_*B*_=1.65 *μ*m, and their respective porosities equal to *P*_*A*_=0.47 and *P*_*B*_=0.67. The parameters *k*=0.56, *ρ*_0_=2,330 kg/m ^3^, and *v*_0_=8,440 m/s were determined by fitting the experimental results as is explained in [23]. In Figure 2, the theoretical (black solid line) and experimental (blue solid line) transmission coefficients (in dB), =*j*_t_/*j*_i_, are plotted as a function of the acoustic phonon frequencies (in GHz). The theoretical transmission spectrum has been modeled using the transfer matrix method and includes the effect of the sample, transducers, and liquid coupling the transducers to the sample. All these components are included writing their corresponding transfer matrix like appearing in (8) but using their respective parameters. The experimental transmission has been measured using a vector network analyzer (VNA) using two piezoelectric transducers according to the experimental setup described in [23]. We can observe that in the range of frequencies reported here, the DBR shows two acoustic gaps, corresponding to the first and second acoustic gap, and centered around ≅0.71 and ≅1.42 GHz, respectively. In the inset, we show the calculated phonon transmittance for frequencies around the zone center of the first minigap. In particular, note that at frequencies *f*_*L*_≅0.54 GHz and *f*_*R*_≅0.88 GHz, corresponding to the edges of the gap, =0 dB. We can observe a noticeable difference in the fundamental bandgap between the experimental and the theoretical spectrum. The experimental bandgap has a depth of approximately 50 dB which is less than the modeled value of approximately 200 dB. However, this is due to the experimental limitation on the setup used, and it is only attributed to the noise of our VNA.

**Figure 3:**
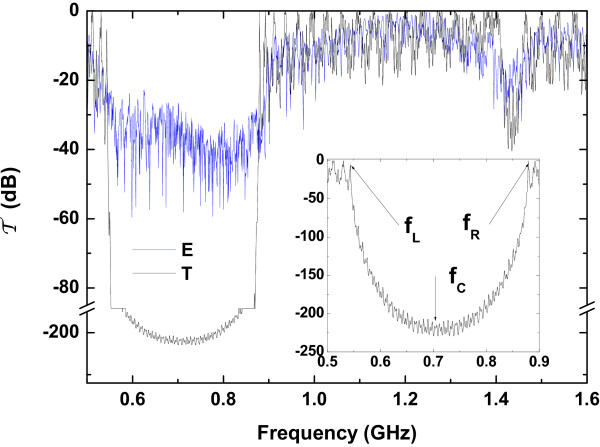
**The vibrational energy density, the one-dimensional stress tensor and the relative displacements.(a)** The vibrational energy density  (in a.u.), **(b)** the one-dimensional stress tensor *σ* (in a.u.), **(c)** and the relative displacements *u* (in a.u.) in the structure, plotted as a function of the normalized distance along the axis of the DBR. The distance is normalized by the factor *lc*=*d*_*A*_+*d*_*B*_.

The behavior of the vibrational energy density  (1) as a function of the normalized distance, along the axis of the DBR, is shown in Figure 3a. We observe that the vibrational energy density is a one piecewise constant function. Let us now investigate the behavior of  at the resonances. At the frequencies *f*_L_≅0.54 GHz (blue solid line) and *f*_R_≅0.88 GHz (red solid line), the vibrational energy density exhibits the maximum value in the center region of the DBR. However, these modes are characterized by complete transmission of the vibrational energy through the system. The one-dimensional stress tensor *σ* and the relative displacements *u* are shown in Figure 3b,c, respectively. In these figures, we have included a schematic representation of the structure, representing with dark (light) regions the layers with low (high) porosity. We observed from Figure 3c that the relative displacement for the mode appearing at *f*_L_≅0.54 GHz (*f*_R_≅0.88 GHz) is localized in the low (high) porosity regions, which is consistent with the agreement used to refer at the valence (dielectric) and conduction (air) band used in semiconductors (photonic crystals). From these figures, it is clearly observed that the phonon modes propagate through the structure.

The phonon modes with energies within the first minigap appear marked by the black solid line in Figure 3b,c. We observe that at the frequency *f*_*C*_≅0.70 GHz, the amplitude of the wave shows an abrupt decay along the axis of the DBR. This qualitative behavior is very similar for the other physical quantities.

#### Tunneling times

The dwell time, *t*_D_, for acoustic phonons inside the DBR is calculated by the following equation [21]:

(36) $$t_{\text{D}}=\frac{H}{j_{\text{t}}},$$

where *H* is the total energy in the interval (0,*L*), being *L* the total length of the DBR. This energy is obtained by integrating Equation 1, i.e.,

(37) $$H=\int_{0}^{L}\mathrm{ℋdz.}$$

The free time, *t*_f_, which is the time associated to the transit of the sound pulse along a distance *L* with velocity, *v*_f_, is simply defined by

(38) $$t_{\text{f}}=\frac{L}{v_{\text{f}}},$$

being *v*_f_ the phase velocity.

**Figure 4:**
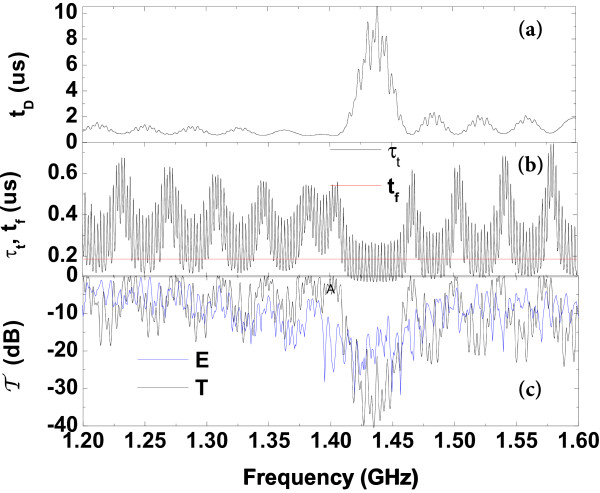
**Tunneling times.(a)** The dwell time *t*_D_ (in *μ*s) and **(b)** the transmission time *τ*_t_ and free time *t*_f_ (in *μ*s) plotted as a function of the acoustic phonon frequencies (in GHz). **(c)** Theoretical and experimental transmission coefficients (in dB) plotted as a function of the acoustic phonon frequencies (in GHz).

In Figure 4, the tunneling times as a function of the phonon frequency (in GHz) are depicted for the acoustic phonons around the second acoustic gap. In Figure 4a, we can observe that the dwell time (36) increases near the gap. The behavior at such frequencies is the same as if the phonon was trapped for a long time in the spatial region occupied for the structure before being transmitted. In Figure 4b, we observe that, in the stop bands, the transmission time (33) is shorter than free time (38), though a large noise is observed due to the coexistence of the liquid modes. The very short transit times in the stop bands are the acoustic equivalent of the Hartman effect [4] of electrons tunneling through potential barriers [8,9].

## Conclusions

In this paper, we studied tunneling times of acoustic phonon packets through a distributed Bragg reflector made of porous silicon layers. Under the assumption that the long-wavelength approximation is valid, and from general principles of symmetry and conservation, we report an explicit demonstration of the equivalence of the transmission and reflection times in the tunneling of long-wavelength phonons. Calculations of the vibrational energy density and the vibrational energy stored within the structure allows a better visualization of the physical phenomena occurring in this system. The description of the stress and strain fields complements the energetic description. We report the possibility that a phenomenon called Hartman effect appears in porous silicon multilayer structures, an acoustic equivalent of Hartman effect of electrons tunneling through potential barriers. The results of this study could be useful for the design of acoustic devices.

## Competing interests

The authors declare that they have no competing interests.

## Authors’ contributions

All authors contributed to the analytical calculations. ZL performed the experimental measurements and fabrication of the porous silicon DBR. All authors contributed to the writing of the manuscript. All authors read and approved the final manuscript.
